# Attachment, Emotion Regulation and Physiological Reactivity in middle-childhood: a pilot study

**DOI:** 10.1192/j.eurpsy.2023.1534

**Published:** 2023-07-19

**Authors:** M. Tironi, S. Charpentier-Mora, F. Bizzi

**Affiliations:** University of Genova, Genova

## Abstract

**Introduction:**

Attachment, as an interpersonal motivational system, is the relational ground that allows emotions to be regulated through action on the Autonomic Nervous System resulting in better psychological outcomes during middle childhood. Emotion dysregulation and autonomic function are often connected to psychiatric symptoms observed in adolescence (e.g. borderline personality disorder traits) and thus middle childhood becomes a critical age for psychopathological trajectories.

**Objectives:**

Examine the relationship between child’s attachment and both psychological and physiological emotional regulation.

**Methods:**

20 children (M_age_ = 10.7, SD = 1.25; 65% males) were recruited from general population. Attachment was measured through the *Child Attachment Interview* (CAI), while emotional regulation was assessed with a multimethod approach, using questionnaires i.e. *How I Feel* (HIF) and *Positive and Negative Affect* (PANAS), and physiological measurements i.e. Heart Rate Variability (ratio between High Frequency and Low Frequency, HF/LF, labeled as the child’s sympathovagal balance) and Heart Rate (Beats Per Minute, BPM, as an index of physiological reactivity).

**Results:**

Statistically significant correlations emerged between the HIF-Control scale and the Mother and Father Dismissal CAI scale, the PANAS-Negative Affect scale and the Mother and Father Preoccupied Anger CAI scale. Moreover, there was a non-significant but moderate effect (*rs* > .30) between HF/LF and the Emotional Openness and the Resolution scales of the CAI, and between BPM and the Emotional Openness scale.

**Conclusions:**

Despite the small sample size, more secure children are able to connect with their emotions and properly use them in solving relational problems. Consequently, they are likely to succeed in understanding and regulating emotions as they manage to find more adaptive strategies their own. On the contrary, the presence of dismissal or anger (insecure attachment) towards parents would appear to lead to higher levels of psycho-physiological dysregulation, a core feature of Borderline Personality Disorder dimensions starting to emerge later in adolescence. Future studies should consider physiological dysregulation as an additional factor linked to psychopathological developmental trajectories.

**Disclosure of Interest:**

None Declared

